# Get in My Belly: Food Preferences Trigger Approach and Avoidant Postural Asymmetries

**DOI:** 10.1371/journal.pone.0072432

**Published:** 2013-08-30

**Authors:** Tad T. Brunyé, Jackie F. Hayes, Caroline R. Mahoney, Aaron L. Gardony, Holly A. Taylor, Robin B. Kanarek

**Affiliations:** 1 Department of Psychology, Tufts University, Medford, Massachusetts, United States of America; 2 Friedman School of Nutrition Science and Policy, Tufts University, Boston, Massachusetts, United States of America; 3 Cognitive Science, United States Army Natick Soldier Research, Development & Engineering Center, Natick, Massachusetts, United States of America; University of California, Merced, United States of America

## Abstract

Appetitive motivational states are fundamental neural and behavioral mechanisms underlying healthy and abnormal eating behavior, though their dynamic influence on food-related behavior is unknown. The present study examined whether personal food-related preferences would activate approach and avoidance systems, modulating spontaneous postural sway toward and away from food items. Participants stood on a balance board that collected real-time data regarding postural sway along two axes (*x, y*) while they viewed a series of images depicting food items varying in nutritional value and individual preferences. Overall, participants showed reliable postural sway toward highly preferred and away from highly non-preferred items. This effect became more pronounced over time; sway along the mediolateral axis showed no reliable variation by preference. Results carry implications for two-factor (homeostatic versus hedonic) neurobehavioral theories of hunger and appetitive motivation, and carry applied clinical implications for the measurement and management of abnormal eating behavior.

## Introduction

The cognitive, affective and behavioral neurosciences have provided strong evidence for bidirectional links between approach and avoidance motivational states and overt (e.g., arm flexion and extension) and covert (e.g., postural sway) indices of motor behavior [1]. Contemporary theory posits that these types of motor behavior reliably reflect the engagement of motivational states that are fundamental to human behavior [2,3]. Research in human feeding behavior has suggested that the approach-related appetitive motivational state is responsible for guiding attention to [4], and directing behavior towards [5] preferred food items, a phenomenon coined “hedonic hunger” [6]. Though there is emerging consensus that experimentally manipulating body posture can influence motivational states (e.g., [7,8]), and viewing aversive images can trigger posterior postural sway (e.g., [9]), relatively sparse research has examined whether posture might reliably index appetitive versus avoidance-related motivations in response to foods. The present study examines this possibility by monitoring anterior (toward) and posterior (away) postural sway and examining whether postural asymmetries can indicate approach and avoidant motivations in response to foods.

### Organizing Behavior via Motivational States

In the late 19^th^ century, Darwin suggested that emotions are adaptive in that they serve to prime or trigger a motivated behavioral response [10]. This early thinking is reflected in contemporary cognitive and neuroscientific theories that emphasize functional links between abstract thought and action. For instance, many recent theories [11,12,13,14,15,16] argue that emotional experience is organized by two basic and distinct motivational systems, appetitive and avoidant. The appetitive system is responsible for approach-related behavior, and is activated in response to pleasant and rewarding stimuli. In contrast, the avoidant (or defensive [3]) system is responsible for motivating active avoidance of negative, aversive stimuli (e.g., disgust, punishment, threat [17]). Behavioral repertoire activated by motivational systems can manifest in several ways, such as through the orienting of attention [11,18,19,4], shifting of body postures [9], arm movements [20,21], and even facilitating or inhibiting the onset of locomotion [22,23] toward and away from stimuli. In each case, the direction of movement toward or away from a stimulus results from an hedonic evaluation along the appetitive-avoidant axis; specifically, approach motivations serve to move people closer to an appetitive stimulus, and avoidance motivations do the opposite [3].

Links between motivational states and covert (e.g., postural sway) and overt (e.g., locomotion) actions manifest through activity in a diverse brain network including the amygdala, nucleus accumbens, ventral striatum, orbitofrontal cortex, and anterior cingulate (for a review, see 24). This brain network provides very rapid assessments of motivational relevance. For instance, event-related potentials (ERPs) suggest that the human brain can attach motivational relevance to pictorial stimuli as early as 125-175 ms following stimulus onset [25,26]. There also appears to be emerging evidence that approach and avoidance systems are differentially reliant on the two brain hemispheres, with left hemisphere regions providing support for approach-related states, and right hemisphere for avoidance-related states [27,28]. For instance, asking people to adopt approach-related postures (e.g., leaning forward with arms extended) activates the left hemisphere, and leaning backward produces the opposite neural pattern [29]. Hence, there is evidence for strong links between activating motivational systems and certain movement types, as well as partially independent neural substrates for approach versus avoidance-related thought and behavior.

### Eating Behavior and Motivational States

Seminal theories explaining human feeding behavior focused on the regulation of homeostatic mechanisms toward controlling food intake. In general, these theories posited a complex regulatory system that precisely maintains both energy balance and body weight (for reviews, see 30,31. More recently, evolutionary (e.g., [32]), neuroscientific (e.g., [33,34]), and behavioral (e.g., [35,36]) evidence supports the utility of complementing homeostasis-based theory with consideration of appetitive motivational states in accounting for real-world human eating behavior (cf. [37]). For this reason, many current theories attempting to explain human food consumption suggest important interactions between homeostatic and hedonic reward-based mechanisms in motivating attention toward palatable foods and driving eating behavior [38.39].

Recent neuroimaging studies have demonstrated a network of brain regions responsible for processing reward across a variety of stimuli, including food, drugs, and alcohol (e.g., [40]). These include the amygdala and orbitofrontal cortex, two densely interconnected regions that appear to be involved in linking an emotionally salient stimulus to memory representations of reward, and predicting the outcomes of consumption, respectively [41]. These two brain regions work in concert with dopaminergic structures including the striatum and anterior cingulate [42]. Indeed dopamine has been implicated as the primary neurotransmitter involved in signaling reward potential in response to food stimuli [43]. Dysregulation of this neural circuitry, and dopaminergic response, have been implicated in obesity; for instance, Wang and colleagues have demonstrated that obese individuals show a more rapid and pronounced dopamine response than normal weight individuals, during exposure to food images [44]. The endogenous opioid system is also thought to interact with this dopaminergic network and appears responsible for the rewarding properties of specifically palatable foods. Activating this reward system through exposure to preferred food items is responsible for facilitating approach-related behavior [37].

### Postural Effects of Motivational State Activation

Bridging the gap between the motivational states and eating literatures, some recent research has demonstrated that overlapping behavioral and neural mechanisms may mediate approach-avoidance behaviors for both food and non-food stimuli. For instance, Gable and Harmon-Jones [45] asked participants to rate their preference for desserts and current hunger, and then exposed them to dessert and neutral pictures while recording brain activity using electroencephalography (EEG). The hungrier the individual was, the greater the left versus right frontal activation while viewing the appetitive desserts versus the neutral images. More recently, Harmon-Jones and colleagues [24] asked participants to adopt forward- or backward-leaning postures while viewing appetitive (dessert) versus neutral stimuli. The approach-related forward lean increased left versus right frontal EEG activity during the viewing of appetitive relative to neutral pictures (see also, 46). Thus, it is becoming clear that body postures can activate lateralized brain regions that are associated with varied motivational states, and these effects can be extended specifically to food-related stimuli.

However, much of the research examining postural variation in response to viewing images selected to promote approach versus avoidance states has found equivocal results. Hillman and colleagues [9] displayed positive, negative and neutral images gathered from a pre-rated image database (IAPS [47]) while monitoring postural sway. Females tended to lean away from unpleasant images (depicting mutilation or attack) relative to pleasant or neutral images, whereas males tended to lean toward pleasant and unpleasant images relative to neutral images. Two additional studies examined postural sway in response to mutilation images and found evidence for reduced anterior–posterior sway, suggesting activation of a defensive freezing posture [48,49]. Similarly, Stins and Beek [50] found no evidence of anterior–posterior sway in response to viewing unpleasant images, but instead found evidence for defensive freezing.

### The Present Study

We propose that equivocal postural effects of positive versus negative image viewing are partially due to the highly subjective nature of hedonic evaluation [51,3]. Relying on standardized images may limit the reliability of hedonic evaluations within image conditions; for instance, whereas one participant might find an image of a family dining at a fast food restaurant as positive, another might find it relatively unpleasant. Similarly, though males tend to rate images of erotica as pleasant, females tend to find them relatively neutral or unpleasant [47]. Thus, while exceedingly negative images depicting mutilation might elicit somewhat reliable postural effects (i.e., freezing or anterior-posterior sway), images categorized as pleasant may not reliably activate approach states. In the present study, rather than assuming that particular images are preferred versus non-preferred, we aimed to understand how individuals’ unique preferences modulate postural responses. This type of design affords a more individualized understanding of whether postural sway might reliably index the activation of approach versus avoidance states. We expected that under these conditions, highly preferred foods would elicit anterior-going sway, and highly non-preferred foods would elicit posterior-going sway. To test this hypothesis, we conducted two experiments; first, a pilot Image Ratings Study aimed at stimulus development, and second, a Main Experiment relating preferences to postural sway.

## Image Ratings Study

The Image Ratings Study was designed to develop a set of pre-rated food images for use in our main experiment. The intention was to select food images that reliably elicit ratings along seven dimensions: healthiness, calorie content, carbohydrate content, fat content, sugar content, protein content, and visual complexity.

## Method

Forty Tufts University undergraduates (20 female, *M*_*age*_ = 19.8) participated for monetary compensation ($20). Each participant rated a total of 495 images gathered using image search engines and existing food image databases. Images represented the five major food groups [52], ranged from whole to prepared, and were modified to conform to 480 x 320 pixels without altering aspect ratio.

Each image was presented one at a time on a computer monitor, and participants rated the image for six nutritional attributes (healthiness, calorie content, carbohydrate content, fat content, sugar content, protein content) and one visual attribute (visual complexity). All ratings were self-paced and completed using a Likert scale ranging from 1 (very low) to 5 (very high).

## Results

Food images varied widely in rated nutritional and visual attributes. Certain images tended to elicit relatively unreliable nutritional attribute ratings, and were removed from further consideration. For instance, mixed nuts and Jello^TM^ elicited low calorie ratings from approximately half of the participants, and high calorie ratings from the others. Similar patterns emerged with the other nutritional attributes.

For visual complexity, we wanted to ensure that any patterns of postural sway in our main experiment could not be confounded by the visual complexity of images. We regressed each nutritional attribute against visual complexity, and selectively removed images that contributed to markedly negative or positive β values. For instance, food items with higher rated sugar content (e.g., candies, cakes) tended to be higher in rated visual complexity (likely due to higher color variation, e.g., jelly beans). Images were removed until visual complexity was no longer significantly related to any single nutritional attribute. This image removal process resulted in 100 remaining images that tended to reliably elicit a wide range of nutritional attribute ratings, as detailed in Table 1. All of these 100 images were incorporated into our Main Experiment.

**Table 1:** Mean, standard deviation, minimum and maximum ratings for each of the seven rated attributes, for the 100 down-selected images for use in our main experiment.

	Healthiness	Calorie Content	Carb. Content	Fat Content	Sugar Content	Protein Content	Visual Complex.
*Mean*	2.92	2.14	2.07	2.88	2.96	1.97	2.52
*St Dev*	1.61	1.51	1.06	1.34	1.18	.79	.57
*Min*	1.09	.56	.93	1.1	1.23	1.18	1.2
*Max*	4.83	4.75	4.22	4.78	4.78	4.38	4.08

### Image Ratings Study Discussion

Our pilot Image Ratings Study examined perceived nutritional and physical attributes for a large set of potential food images, resulting in a set of images that reliably elicited a range of nutritional ratings without confounding visual complexity.

## Main Experiment

The main experiment examined anterior–posterior postural sway in response to viewing the 100 images selected in our Image Ratings Study. We specifically asked whether participants’ preferences for particular food types, and individual differences in eating behavior, would predict the magnitude and time course of postural responses to image viewing. The extant literature suggests that approach-related motivational states trigger covert postural biases toward (approach) and away (avoidance) from items associated with varied hedonic evaluations. In line with this work, we predicted that highly preferred food items will elicit anterior sway (i.e., sway toward an image) and non-preferred food items will elicit posterior sway (i.e., sway away from an image). We expected these effects to manifest relatively quickly, as suggested by psychophysiological evidence pointing to a rapid time course of activating motivational attributes to perceived images. Finally, we asked whether the magnitude of individuals’ anterior or posterior sway in response to images would be predicted by participant demographics, and state and trait measures of eating behavior. We included these measures to test whether trait-based eating behavior (e.g., restraint, dieting, binging) and state-based hunger would modulate some relationships between preferences and postural sway. Specifically, we hypothesized that higher dietary restraint might increase approach-related anterior sway in response to preferred foods; this hypothesis is based on research demonstrating restrained eaters show selective attention toward hedonically relevant food items [53]. We also hypothesized that state-based hunger might increase anterior sway in response to preferred foods; this hypothesis is based on research demonstrating that short-term food deprivation can increase approach-related reaction toward preferred food items [54,55].

### Method

#### Ethics Statement

The research was approved by the Tufts University Social, Behavioral, and Educational Research Institutional Review Board (SBER IRB). Participants provided their written informed consent to participate in the study.

#### Participants

One Hundred Tufts University undergraduates (65 female, *M*_*age*_ = 19.9, *M*_BMI_ = 22.5) participated for monetary compensation ($20).

#### Materials

**Figure 1:**
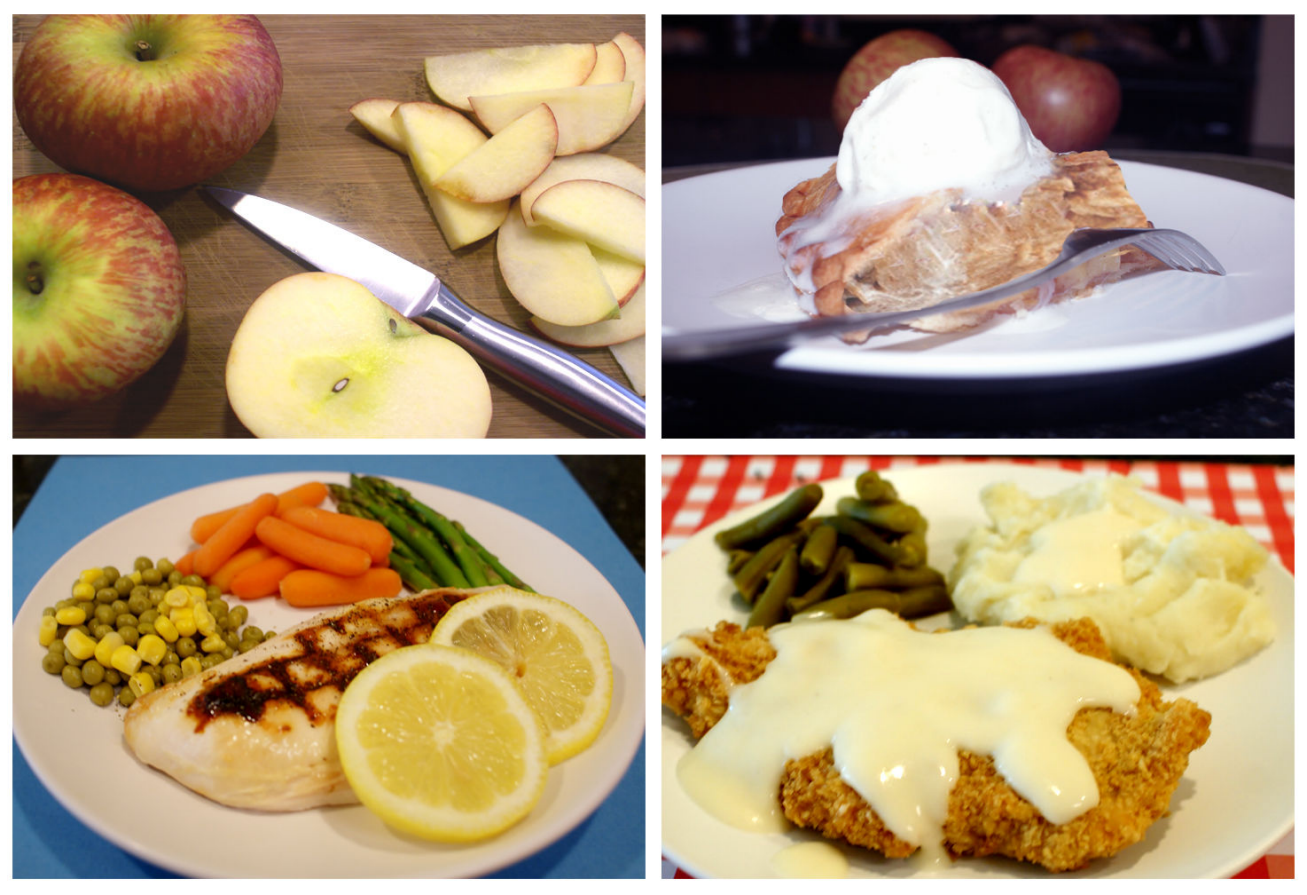
Example food images from the Main Experiment, ranging in nutritional value. Depicted are sliced apples (A), apple pie (B), grilled chicken and vegetables (C), and fried chicken and vegetables (D). Note: Images shown here are for illustrative purposes only due to copyright restrictions.

We used the 100 food images down-selected from the Image Ratings Study and described in Table 1. Example images are depicted in Figure 1. Participants stood on a Wii balance board that collected center of pressure (COP) data along two (*x*, *y*) axes at 16Hz. The Wii balance board is considered a valid apparatus for assessing standing balance, with adequate reliability and sensitivity when compared to professional grade force platforms [56,57]. The device samples center of pressure data at 16Hz (approximately every 62.5 ms). The Wii board has a standing surface of 45 x 26.5 cm, and it was placed on a hard flat surface in the center of the laboratory.

A 17″ LCD computer monitor was attached to the top of a large Manfrotto^TM^ 546B tripod that adjusted vertically to match each participant’s eye height; the monitor was positioned directly in front of the participant, 48″ from the eye. Several demographic variables were collected, including age, height, weight, and gender (BMI was calculated). We also administered a series of questionnaires related to eating behavior. These included the Three-Factor Eating Questionnaire (TFEQ; [58]), the Satiety Labeled Intensity Magnitude (SLIM; [59]), and questions aimed at assessing overall eating habits (poor to excellent), current weight goals (lose, maintain, gain), and history of weight loss programs, and two months’ history of weight fluctuation (lost, maintained, gained).

#### Procedure

Informed consent was gathered and participants stood on the balance board; an automated calibration process lasting approximately 5 sec established a neutral center of pressure for each individual. Participants then began viewing the 100 images in random order, one at a time for 3 sec each, centered on the computer monitor, and each immediately preceded by a 3 sec fixation cross. Participants were then seated at a desktop and we again presented the 100 images and asked them to rate their liking of each food item using a 5-point Likert scale ranging from highly disliked (highly non-preferred) to highly liked (highly preferred). They then completed the demographic and eating behavior questionnaires, and were thanked for their time and dismissed from the study.

## Results

Raw center of pressure (COP) data are referenced to a standardized 2x2 coordinate grid with limits of -1 and +1 along the mediolateral and anterior–posterior axes; in other words, extreme anterior sway would produce a maximum COP of +1, and extreme posterior sway a -1. To promote comparison to extant literature (e.g., [57]) these coordinate data were converted to centimeters with reference to the overall balance board dimensions (26.5h x 45w cm).

To account for postural drift during standing over prolonged time periods (i.e., [60]), each trial’s raw center of pressure (*x, y*) data were referenced to a 500 ms pre-stimulus window. The 3000 ms image time was collapsed to 6 bins (500 ms each); data from each time bin are detailed in Table 2. To examine whether participants’ unique preferences for food items modulate postural sway toward or away from the item, we parsed food items by individual participant preference ratings, using three categories: non-preferred, no strong preference, or highly preferred. The total number of trials was 10,000; when parsed into categories, non-preferred contained 1725 trials, no strong preference contained 6208 trials, and highly preferred contained 2065 trials. Critically, these proportions were highly similar across participants, and no single participant was missing data from any preference category. Data were subjected to a 3 (Preference) x 6 (Time) repeated-measures analysis of variance (ANOVA).

**Table 2:** Time-binned mean and standard error center of pressure (COP) in cm along the anterior–posterior axis for each of the three food preference conditions, and fixation.

		Time Bin 1	Time Bin 2	Time Bin 3	Time Bin 4	Time Bin 5	Time Bin 6
*Non-Preferred*	*Mean*	.042	-.053	-.256	-.369	-.413	-.346
	*Std Err*	.063	.076	.098	.095	.103	.113
*No Strong Preference*	*Mean*	.018	-.026	-.154	-.204	-.137	-.089
	*Std Err*	.024	.033	.046	.047	.054	.058
*Highly Preferred*	*Mean*	.052	.163	.191	.314	.442	.561
	*Std Err*	.056	.101	.117	.156	.165	.181
*Fixation Window*	*Mean*	.001	.002	.001	.001	-.0001	-.0001
	*Std Err*	.0002	.001	.001	.001	.001	.002

Questionnaires were scored in accordance with standard procedures. The TFEQ was parsed into three subscales corresponding to restraint, disinhibition, and trait hunger. The SLIM was scored as positive (currently satiated) versus negative (currently hungry) deviation from center (zero). The remaining questions were coded to reflect each option; overall eating habits ranged from excellent (1) to very poor (5), current weight goals were coded as lose (-1), maintain (0), and gain (+1), history of weight loss programs was coded as yes (1) or no (0), and recent history of weight loss fluctuation was coded as lost (-1), maintained (0), and gained (+1).

### Preferences and Nutritional Content

A stepwise linear regression examined whether the rated (see Image Ratings Study) healthiness or nutritional content (calories, carbohydrates, fats, sugars, protein) of foods predicted preference ratings. The regression model showed that healthiness was the only factor independently predictive of preference ratings, *F*(1, 9748) = 922.51, *p* < .001, *R* = .29. Overall, participants tended to give higher preference ratings to foods with higher rated healthiness (β_std_ = .29, *p* < .001).

### Postural Sway Analyses

**Figure 2:**
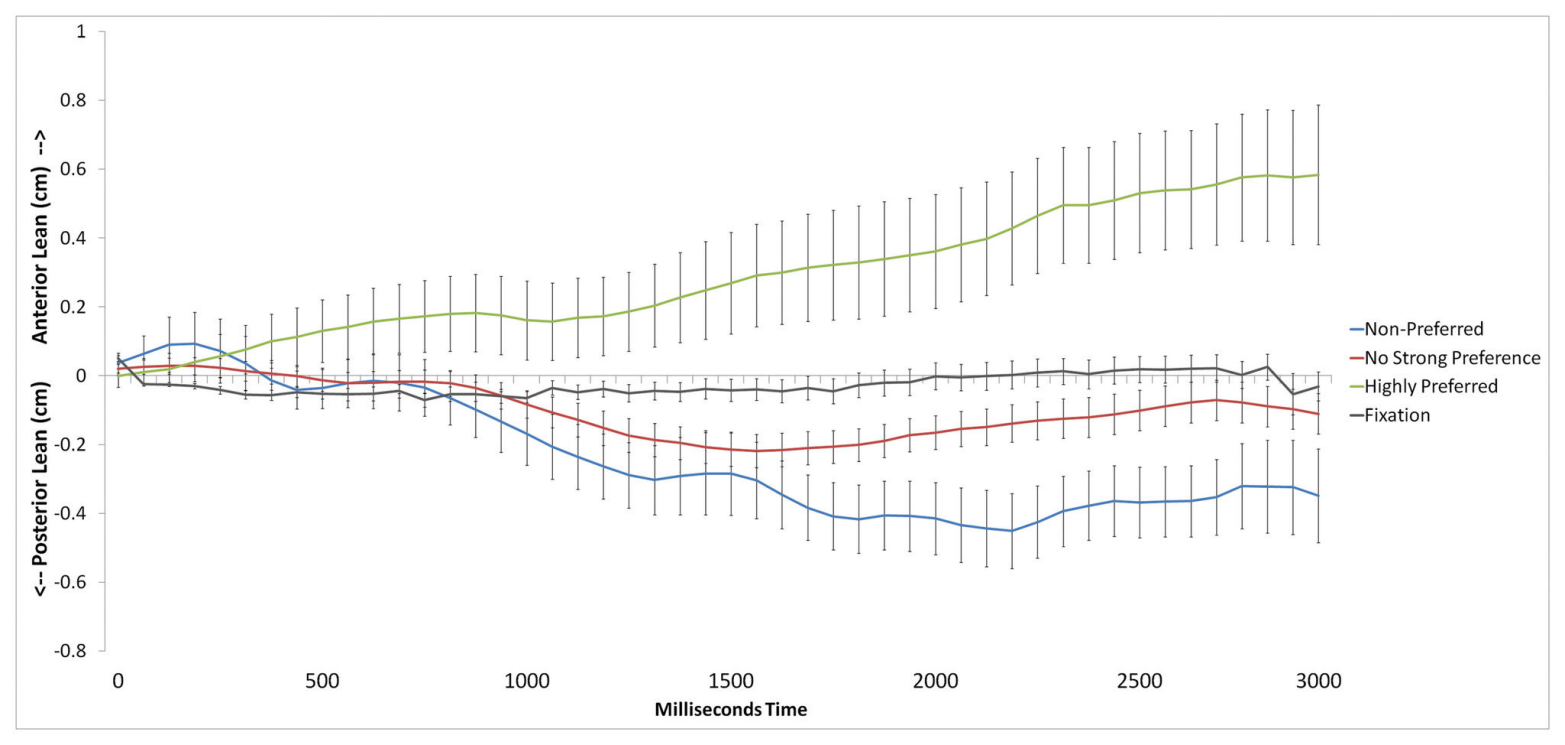
Anterior (forward) versus posterior (backward) postural sway as a function of food item preferences and time relative to image onset. Fixation (referenced to 500ms post-onset baseline) provided for visual comparison.

We found evidence that individual preferences modulated anterior–posterior postural sway, as shown in Figure 2. The ANOVA demonstrated main effects of Preference, *F*(2,198) = 10.27, *p* < .001, η^2^ = .06, and Time, *F*(5,495) = 2.84, *p* = .015, η^2^ < .01. These effects were qualified by an interaction between Preference and Time, *F*(10,990) = 10.16, *p* < .001, η^2^ = .02. Simple effects ANOVAs tested for Preference effects within each Time bin. Time bins 1 (0–500 ms) and 2 (500–1000 ms) did not reach significance (*p* = .89, *p* = .09, respectively). Effects were found in Time bins 3 (1000–1500 ms, *F*(2,198) = 6.12, *p* < .01, η^2^ = .06), 4 (1500–2000 ms, *F*(2,198) = 10.46, *p* < .001, η^2^ = .10), 5 (2000–2500 ms, *F*(2,198) = 14.14, *p* < .001, η^2^ = .13), and 6 (2500–3000 ms, *F*(2,198) = 13.31, *p* < .001, η^2^ = .12). Participants showed reliable sway toward high preference items and posterior sway in the other two conditions. To ensure that our results were not driven by the unequal number of trials per condition, follow-up analyses using a random sample of trials for each condition equal to the number of trials in the smallest condition (1725), showed maintained significance in the Preference x Time interaction (*p* < .001), and in Time bins 3 (*p* = .02), 4 (*p* < .01), 5 (*p* < .001), and 5 (*p* < .01) (cf. [61]).

To specifically examine these effects, we conducted pair-wise comparisons within time bins 3-6 using a Bonferroni correction term (α_crit_ = .017). Within time bin 3, the Highly Preferred condition showed more anterior lean relative to the No Strong Preference, *t*(99) = 2.82, *p* < .01, d = .42, and Non-Preferred, *t*(99) = 2.79, *p* < .01, d = .41, conditions (No Strong Preference vs. Non-Preferred, *p* = .38). Within time bin 4, the Highly Preferred condition showed more anterior lean relative to the No Strong Preference, *t*(99) = 3.14, *p* < .01, d = .42, and Non-Preferred, *t*(99) = 3.71, *p* < .001, d = .41, conditions (No Strong Preference vs. Non-Preferred, *p* = .13). Within time bin 5, the Highly Preferred condition showed more anterior lean relative to the No Strong Preference, *t*(99) = 3.38, *p* < .001, d = .52, and Non-Preferred, *t*(99) = 4.35, *p* < .001, d = .64, conditions; the Non-Preferred also showed more posterior lean relative to the No Strong Preference condition, *t*(99) = 2.44, *p* < .017, d = .35. Finally, within time bin 6, the Highly Preferred condition showed more anterior lean relative to the No Strong Preference, *t*(99) = 3.53, *p* < .001, d = .54, and Non-Preferred, *t*(99) = 4.11, *p* < .001, d = .62, conditions (No Strong Preference vs. Non-Preferred, *p* = .04, d = .30).

An ANOVA examining mediolateral (left–right) sway showed a main effect of Time, *F*(5,495) = 2.91, *p* = .01, η^2^ < .01, with participants tending to lean slightly more rightward over time. There was no effect of Preference (*p* = .71), and there was no interaction (*p* = .11).

A linear regression demonstrated that demographics (age, gender, BMI), eating behavior, weight goals and eating habits did not reliably predict the magnitude of individuals’ anterior/posterior responses to preferred versus non-preferred food items, *F*(8,98) = 1.87, *p* = .07, *R* ^2^_max_ = .14.

An exploratory ANOVA showed that median splits by rated healthiness did not reliably predict anterior–posterior sway, though participants did show numerical evidence of anterior sway toward relatively healthy, and posterior sway away from relatively unhealthy, foods. This pattern corresponds to regression results.

## Discussion

Our main experiment examined postural effects of viewing food images towards which individual participants showed low versus high preference. We hypothesized that food items with higher preference ratings would elicit anterior postural sway, whereas non-preferred items would elicit posterior postural sway. This pattern was expected to manifest within a few seconds of viewing a food-related image. We also hypothesized that the extent of these effects might be predicted by individual differences in eating behavior, demographics, or state hunger. Results provide partial support for these hypotheses. First, we found strong evidence that participants indeed leaned toward and away from preferred and non-preferred food items, respectively. Second, the time course of this effect was similar across the two sway directions (anterior/posterior), with conditions first diverging at approximately 1000-1500ms post stimulus onset, and then increasingly diverging toward the end of the stimulus window.

We found no evidence that individual differences in demographics or eating behavior (including state hunger) reliably predicted the magnitude of participants’ postural sway. Indeed the present effects were consistent across individuals with varied demographics and levels of eating restraint, disinhibition, and trait and state hunger, suggesting that postural indices of food-related approach and avoidance are both robust and reliable across individuals with varied eating, dieting, and weight management profiles. These results suggest that restraint- and hunger-related attentional biases identified earlier work do not translate to pronounced whole-body motor responses toward preferred food items. In other words, though visual attention might selectively bias restrained or hungry individuals toward preferred food items [54,53,55], top-down control mechanisms might prevent them from initiating motor responses toward these foods (i.e., [62]). Future work might attempt to disentangle attentional versus motor responses by employing concurrent measures, such as using eye tracking to predict the extent of postural sway.

## General Discussion

A diverse neural network has been implicated in linking the activation of motivational states and the instantiation of covert and overt motor behavior, including the amygdala, nucleus accumbens, ventral striatum, orbitofrontal cortex, and anterior cingulate. This network affords rapid motivational evaluations of perceived objects [25] and the activation of approach and avoidance states that facilitate certain types of motor behavior [24]. Recent empirical work has demonstrated that these motor behaviors manifest at many scales (e.g., arm movements, postural sway, locomotion), reinforcing theoretical advances that posit reciprocal links between emotions and motivated behavioral responses [13,14,16]. The present findings support this notion by uniquely demonstrating that activating motivational systems by viewing preferred versus non-preferred foods reliably alters whole-body postural sway.

What neural systems might underlie the present effects? Kringelbach [63] proposed a neurocognitive model of the interactions between perceiving foods, activating evaluating systems, and guiding behavior. In this model, sensory information including visual, olfactory, and gustatory representations are processed in primary sensory cortices where stimulus identity is decoded. Stimulus identity spontaneously activates multi-modal representations in the posterior orbitofrontal cortex that incorporate prior experiences with the perceived food item. Reward value representations are activated in the relatively anterior portions of the orbitofrontal cortex, where hedonic value is assigned and behavioral responses (i.e., approach or avoid [64]) are motivated in conjunction with the anterior cingulate and dorsolateral prefrontal cortex. These very same brain regions have been implicated in reward processing with drugs and alcohol [40]), are involved in dopaminergic response to subjective liking [43], and have been strongly implicated in the activation and active control of attention and behavior [65,66,67,42].

Dysregulation of neural circuitry including the orbitofrontal cortex and anterior cingulate is associated with several disorders including attention deficit hyperactivity disorder (ADHD; [68]), and drug addiction [69]. It may be the case that abnormally increased hedonic value of perceived rewards (drugs, foods, etc), and thus increased dopaminergic response, are responsible for some compulsive eating and drug use [70]. In normal eating behavior, appetitive preferences tend to include relatively healthy foods [71] whereas abnormal eating behavior, such as in obesity, shows preference shifts towards relatively unhealthy food options [72]. Thus, behavioral therapies for the treatment of obesity, such as the Diabetes Prevention Program [73], train strategies aimed at altering food preferences through increasing self-monitoring and the control of behavioral responses to food stimuli. We propose that monitoring postural shifts relative to preferred foods, and evaluating those preferences for health value, could provide a powerful clinical tool for assessing therapeutic program efficacy.

### Limitations

The present college student sample was characterized by relatively healthy eating habits, a restricted BMI range, and low dieting rates. Only 14 of our 100 participants were overweight (BMI range 25-29.9), and four were obese (BMI > 30), according to current recommendations; further, only four participants reported a history of weight loss program enrollment. Similarly, no participants noted anything more than slight hunger during the experimental session. A broader range of eating habits, BMI, dieting history, and state hunger might show value toward predicting postural sway in response to foods. Future research will sample participants from a wider demographic to assess the universality of preference influences on postural sway. A second limitation is related to the limited sensory experience on behalf of participants. Food intake is a multi-sensory experience involving not only vision but also taste, smell, sound, and texture; though we did find compelling results using only vision, more immersive sensory experiences that involve actual food exposure might be expected to elicit more robust postural effects. As Kringelbach [63] notes, the extent of neural response to food exposure might be positively predicted by the nature and extent of sensory input.

## Conclusions

Our approach was particularly unique in that it assessed subjective assessments of food items, a highly individualized approach to understanding links between perception, brain and behavior. Food intake relies on individuals perceiving available foods, evaluating them for hedonic value, and selecting a behavior. Hedonic appraisal is highly subjective and driven by knowledge and experience, and is considered fundamental in the control of eating [51.74]. The present work suggests that spontaneous and covert postural shifts toward foods with high personal hedonic value may potentiate behavioral movements toward perceived foods, perhaps resulting in consumption (cf. [37]). This finding supports emerging theory positing links between motivational systems and behavior, and carries clinical implications for the measurement and management of abnormal eating behavior.
